# Efficacy of antioxidant in idiopathic pulmonary fibrosis: A systematic review and meta-analysis

**DOI:** 10.17179/excli2016-619

**Published:** 2016-11-07

**Authors:** Amit D. Kandhare, Anwesha Mukherjee, Pinaki Ghosh, Subhash L. Bodhankar

**Affiliations:** 1 Department of Pharmacology, Poona College of Pharmacy, Bharati Vidyapeeth Deemed University, Erandwane, Paud Road, Pune-411 038, India

**Keywords:** antioxidant, meta-analysis, idiopathic pulmonary fibrosis, vital capacity, carbon monoxide diffusing capacity

## Abstract

Idiopathic pulmonary fibrosis (IPF) is a chronic oxido-inflammatory disorder of the lung. Oxidative stress is widely recognized as a central feature of IPF. Antioxidant therapy has been proposed as an effective treatment for IPF. An array of clinical trials describing the therapeutic impact of these drugs have been reporting albeit with conflicting evidence points. We performed a meta-analysis of trials in which efficacy of antioxidant therapy was compared with control in IPF. Systematic literature search was conducted in PubMed, EMBASE, the Cochrane Library, CPCI-S (Conference Proceedings Citation Index-Science), ICTRP (International Clinical Trials Registry Platform), and Google Scholar till June 2016 by two independent researchers. Various outcomes such as changes in pulmonary function tests (change in vital capacity [ΔVC], change in forced vital capacity [ΔFVC], change in percentage of predicted vital capacity [Δ%VC], and change in percentage of predicted carbon monoxide diffusing capacity [Δ%DLco]), changes in 6 minutes walking test distance (Δ6MWT), rates of adverse events, and rates of death, were included. All statistical analyses were performed using RevMan V.5.3. Twelve studies (n = 1062) were identified that used antioxidants (N-acetylcysteine and lecithinized superoxide dismutase) as a treatment for IPF. Overall, there was no association of antioxidant therapy with ΔFVC (SMD = 0.27, 95% CI:-0.07 to 0.61; P = 0.12), ΔFVC (%) (SMD = -0.10, 95% CI:-0.56 to 0.36; P = 0.66) and 6MWT (SMD = -0.04, 95% CI:-0.11 to 0.20; P = 0.59) in IPF patients. However, combined antioxidant therapy was found to be associated with %VC (SMD = 0.37, 95% CI: 0.09 to 0.64; P = 0.008) and Δ%DLco (SMD = 0.15, 95% CI: 0.00 to 0.29; P = 0.05) in IPF patients. Strong evidence was obtained that the antioxidants increased adverse effects adverse events (OR = 1.56, 95% CI: 0.75 to 3.24; P = 0.23) but it did not associate mortality (OR = 0.96, 95% CI: 0.44 to 2.11; P = 0.92). The use of significant clinical heterogeneity, low statistical power, high dropout rates, duration of follow-ups, and dosing regimens of antioxidant agents. Combined antioxidant therapy seems to be a safe and effective therapy for IPF patients which provides a more beneficial effect in terms of VC, and DLco rather than monotherapy. Further randomized controlled trials with homogeneous outcome measures are needed.

## Introduction

Idiopathic pulmonary fibrosis (IPF) is a chronic progressive, debilitating interstitial lung disease with unknown etiology. Diffused scarring (fibrosis) and mild inflammation are characteristic features of IPC culminating to a gradual worsening of lung capacity (Raghu et al., 2011[27]). In the United States, its prevalence ranges between 14 and 27.9 cases per 100,000 population using narrow case definitions, and 42.7 and 63 per 100,000 population using broad case definitions. In Europe, it ranges from 1.25 to 23.4 cases per 100,000 population (Raghu et al., 2015[28], 2016[25]), whereas 494.5 per 100,000 individuals was the prevalence among the Medicare-eligible population in 2011 (Raghu et al., 2014[26]). In the USA the annual incidence rate was estimated at 6.8-8.8 per 100,000 population using narrow case definitions and 16.3-17.4 per 100,000 population using broad case definitions whereas in Europe it ranged between 0.22 and 7.4 per 100,000 population (Nalysnyk et al., 2012[21]). Mainly males over 60 years of age are more affected by IPF than females with peak prevalence in the eighth decade (du Bois, 2012[12]; Gribbin et al., 2006[13]). 

The clinical manifestations of IPF include shortness of breath and a cough with significant impairment in health-related quality of life (HRQoL). Cigarette smoking, gastroesophageal disease, environmental exposure, and possibly infective agents such as influenza, Epstein-Barr virus and hepatitis are some of the factors that are associated with the IPF (American Thoracic Society, 2000[1]). HRQoL of IPF patients deteriorates rapidly with an approximate survival rate of 2 to 5 years. According to THIN's database cohort, 180 per 1000 person-years (95% CI 164 to 198 per 1000 person-years) was the mortality rate for people with IPF (Gribbin et al., 2006[13]). There are only a few effective therapies available, and none offers a cure. Moreover, the mortality rate is high warranting the need of new treatments for IPF (Bando et al., 2010[3]; Behr et al., 2016[5]; Homma et al., 2012[14]; Martinez et al., 2014[20]; Tomioka et al., 2005[36]).

An array of treatments such as anti-inflammatory therapy with corticosteroids or immunosuppressants have been evaluated for use in patients. However, this therapy fails to significantly improve the survival time of patients with IPF (Lee et al., 2011[18]; Pinheiro et al., 2008[23]; Taskar and Coultas, 2006[35]; Xaubet et al., 2003[37]). Other pharmacotherapies such as nintedanib, etanercept, warfarin, Gleevec, and bosentan have limitations owing to their adverse effects (Luppi et al., 2012[19]). Various working groups which formulate treatment algorithms have found insufficient evidence to recommend any of them as full fledged clinical practice guidelines (Raghu et al., 2011[27]). In 2011, Pirfenidone was approved by the European Medicines Agency for the treatment of IPF (Jiang et al., 2012[16]; Taniguchi et al., 2010[34]). However, a fraction of patients discontinued the treatment pertaining to side effects (Cottin and Maher, 2015[10]).

Lungs are the primary organs frequently exposed to higher oxygen tension. Several clinical and experimental studies have documented that oxidative stress caused cell membrane destruction via generation of free radical peroxidation products leading to pulmonary fibrosis (Beeh et al., 2002[4]; Cantin et al., 1989[7]). Furthermore, a considerable body of evidence suggests that antioxidant supplementation could be a potentially useful treatment for IPF. Various clinical studies have reported the efficacy of antioxidant treatment (such as N-acetylcysteine (NAC) and lecithinized superoxide dismutase (PC-SOD)) for IPF (Bando et al., 2010[3]; Behr et al., 2009[6], 2016[5]; Homma et al., 2012[14]; Huang et al., 2015[15]; Kamio et al., 2014[17]; Martinez et al., 2014[20]; Okuda et al., 2016[22]; Raghu et al., 2012[24]; Sakamoto et al., 2013[30], 2015[31]; Tomioka et al., 2005[36]). Several studies found that NAC treatment had a beneficial effect on lung function in IPF patients and significantly delayed disease progression (Behr et al., 2009[6]; Homma et al., 2012[14]; Huang et al., 2015[15]; Okuda et al., 2016[22]; Sakamoto et al., 2013[30], 2015[31]) whereas few studies reported that NAC treatment had no benefit and did not significantly improve the lung function of patients with IPF (Bando et al., 2010[3]; Behr et al., 2016[5]; Martinez et al., 2014[20]; Raghu et al., 2012[24]). A study by Tomioka et al. (Tomioka et al., 2005[36]) showed that N-acetylcysteine aerosol treatment might delay disease progression, but did not improve pulmonary function or quality of life (QoL). However, PC-SOD treatment had a beneficial effect on serum markers in IPF patients with severe respiratory dysfunction (Kamio et al., 2014[17]). As low statistical power may limit the interpretability of the findings, the results of all of these trials (Bando et al., 2010[3]; Behr et al., 2009[6], 2016[5]; Homma et al., 2012[14]; Huang et al., 2015[15]; Kamio et al., 2014[17]; Martinez et al., 2014[20]; Okuda et al., 2016[22]; Raghu et al., 2012[24]; Sakamoto et al., 2013[30], 2015[31]; Tomioka et al., 2005[36]) should be interpreted with caution. In view of this conflicting evidence landscape, it is important to create an empirical evidence framework to underline the degree of therapeutic benefit of antioxidant therapy in IPF patients. Hence, the aim of the present study was to conduct systematic review and meta-analysis of effectiveness and potential side effects of antioxidants for the treatment of IPF to develop a robust evidence base for robust recommendations for clinical decisions.

## Methods

### Data sources and selection criteria

This systematic review and meta-analysis were conducted as per Preferred Reporting Items for Systematic Reviews and Meta-Analyses (PRISMA) guidelines (Stewart et al., 2015[32]). The following electronic databases were searched: PubMed, EMBASE, the Cochrane Library, CPCI-S (Conference Proceedings Citation Index-Science), ICTRP (International Clinical Trials Registry Platform), and Google Scholar were searched in June 2016. Two independent reviewers (ADK and AAM) conducted an abstract review of all records. The following keywords were used in the search strategy: Diverse anti-oxidative substances, such as vitamin C, vitamin E, allopurinol, b-carotene, selenium, and methionine, acetylcysteine, N-acetylcysteine, NAC, idiopathic pulmonary fibrosis, and IPF. 

Inclusion criteria were as follows: studies that compared an N-acetylcysteine-treated group with a control group for the treatment of IPF; studies that reported outcome measures, including changes in pulmonary function tests (change in vital capacity [ΔVC], change in forced vital capacity [ΔFVC], change in percentage of predicted vital capacity [Δ%VC], and change in percentage of predicted carbon monoxide diffusing capacity [Δ%DLco]), changes in 6 minutes walking test distance (Δ6MWT), rates of adverse events, and rates of death; and filtered by articles published in English and Humans.

Exclusion criteria were: experimental studies; mechanistic (association) studies; Letters/reviews/editorials, commentary, animal studies, in-vitro studies, Case series (sample size <10 patients), case reports, pharmacodynamic/pharmacokinetic studies and studies with full-text published in a language other than English.

### Quality assessment of the articles

The quality of each study included in the analysis was assessed using the Cochrane Risk of Bias Tool for systematic reviews of interventions (version 5.0.1) (Cochrane, 2008[9]) and also using the Downs and Black critical appraisal tool. This validated Cochrane Risk of Bias tool consisted of the following six categories: (1) random sequence generation; (2) allocation concealment; (3) blinding of participants; (4) incomplete outcome data; (5) selective outcome reporting; and (6) other bias. Each category was scored as high, uncertain, or low ROB. Two independent reviewers performed the quality assessment and disagreements on scores were resolved through discussion.

### Data analysis

The standard mean difference (SMD) was used with 95% confidence intervals (CI) and standard deviation (SD). A meta-analysis was conducted with simultaneous use of random-effect models. All statistical analyses were performed using RevMan V.5.3 (Cochrane, Oxford, UK) software. The meta-analysis included data from parallel-group design studies. Since the data used for the meta-analysis were continuous variables such as ΔVC, ΔFVC, Δ%VC, Δ%DLco and Δ6MWT, the standardized mean difference (SMD) and 95% CI were used for meta-analysis. Statistical analysis for dichotomized outcomes (rates of adverse events, and rates of death) was performed using odds ratio (OR) and 95% CI. Heterogeneity of the included studies was tested with the Higgins I^2 ^test, and meaningful heterogeneity was determined by 50 % of the I^2^ value. When the I^2^ value was >50, a random-effect model was used for the meta-analysis. 

## Results

### Summary of included studies

**Figure 1:**
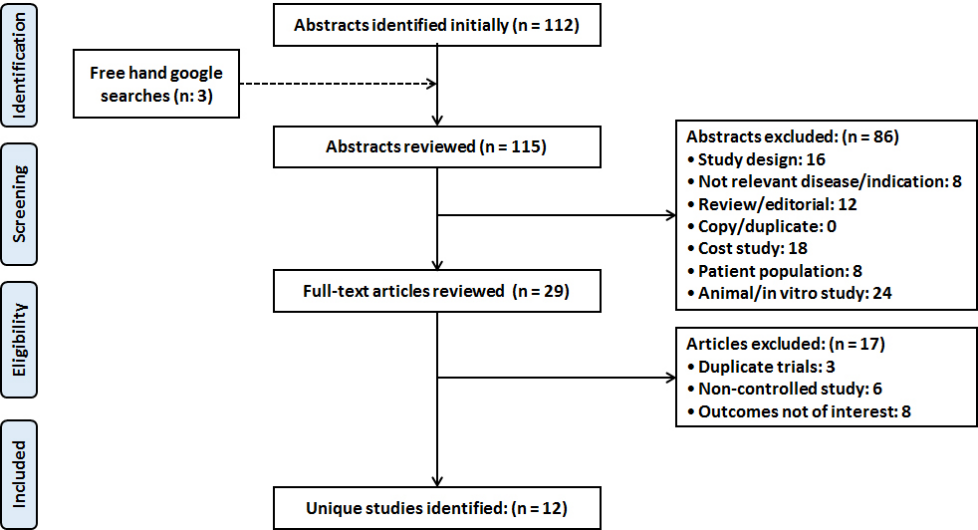
PRISMA flow diagram depicting the selection of studies for systematic review and meta-analysis

As shown in Figure 1(Fig. 1) (PRISMA flow chart), the database searches initially yielded 115 results. After screening, 86 of these (study design (16), not relevant disease/indication (8), review/editorial (12), cost study (18), patient population (8) and animal/in vitro study (24)) were excluded. Further, seventeen more studies were deemed irrelevant, based on the title or abstract, and were also excluded. Of the remaining twelve studies (Bando et al., 2010[3]; Behr et al., 2009[6], 2016[5]; Homma et al., 2012[14]; Huang et al., 2015[15]; Kamio et al., 2014[17]; Martinez et al., 2014[20]; Okuda et al., 2016[22]; Raghu et al., 2012[24]; Sakamoto et al., 2013[30], 2015[31]; Tomioka et al., 2005[36]) were included for qualitative and quantitative analysis.

The primary findings from the included studies are summarized in Table 1(Tab. 1). Eight studies were conducted in Japan (Bando et al., 2010[3]; Homma et al., 2012[14]; Huang et al., 2015[15]; Kamio et al., 2014[17]; Okuda et al., 2016[22]; Sakamoto et al., 2013[30], 2015[31]; Tomioka et al., 2005[36]), three in multi-countries (Behr et al., 2009[6], 2016[5]; Raghu et al., 2012[24]) and one in United States (Martinez et al., 2014[20]). Only two types of antioxidants (N-Acetylcysteine (NAC) (Bando et al., 2010[3]; Behr et al., 2009[6], 2016[5]; Homma et al., 2012[14]; Huang et al., 2015[15]; Martinez et al., 2014[20]; Okuda et al., 2016[22]; Raghu et al., 2012[24]; Sakamoto et al., 2013[30], 2015[31]; Tomioka et al., 2005[36]) and lecithinized superoxide dismutase (PC-SOD) (Kamio et al., 2014[17]) were reported in these studies. Six studies (Bando et al., 2010[3]; Homma et al., 2012[14], Okuda et al., 2016[22]; Sakamoto et al., 2013[30], 2015[31]; Tomioka et al., 2005[36]) had inhalation as a route of administration of NAC whereas five studies (Behr et al., 2009[6], 2016[5]; Huang et al., 2015[15]; Martinez et al., 2014[20]; Raghu et al., 2012[26]) indicated NAC as an oral treatment and in only one study (Kamio et al., 2014[17]) PC-SOD was administered intravenously.

### Characteristics of the studies included

Table 1(Tab. 1) shows the study characteristics. There was a total of 1,062 enrolled patients, of which 530 (49.9 %) patients received antioxidant treatment, whereas remaining were on placebo. Eight studies were randomized controlled trials (seven were double blinded (Behr et al., 2009[6], 2016[5]; Homma et al., 2012[14]; Huang et al., 2015[15]; Kamio et al., 2014[17]; Martinez et al., 2014[20]; Raghu et al., 2012[24]) and one was open arm (Tomioka et al., 2005[36])); while three were retrospective studies (Bando et al., 2010[3]; Sakamoto et al., 2013[30]), one had a single-arm prospective clinical trial (Okuda et al., 2016[22]) and case-control trial study (Sakamoto et al., 2015[31]), respectively.

Health status of IPF patients was determined by ΔFVC, Δ%VC, Δ%DLco, and Δ6MWT. The change in percentage of predicted vital capacity [Δ%VC] was measured in three studies (Behr et al., 2009[6]; Homma et al., 2012[14]; Tomioka et al., 2005[36]), change in forced vital capacity [ΔFVC] was measured in eleven studies (Bando et al., 2010[3]; Behr et al., 2016[5]; Homma et al., 2012[14]; Huang et al., 2015[15]; Kamio et al., 2014[17]; Martinez et al., 2014[20]; Okuda et al., 2016[22]; Raghu et al., 2012[24]; Sakamoto et al., 2013[30], 2015[31]; Tomioka et al., 2005[36]) whereas seven studies (Bando et al., 2010[3]; Behr et al., 2009[6], 2016[5]; Huang et al., 2015[15]; Martinez et al., 2014[20]; Raghu et al., 2012[24]; Tomioka et al., 2005[36]) measured change in percentage of predicted carbon monoxide diffusing capacity [Δ%DLco] and five studies (Behr et al., 2016[5]; Huang et al., 2015[15]; Martinez et al., 2014[20]; Raghu et al., 2012[24]; Tomioka et al., 2005[36]) measured changes in 6 minutes walking test distance (Δ6MWT). Adverse events were reported by ten studies (Bando et al., 2010[3]; Behr et al., 2016[5]; Homma et al., 2012[14]; Huang et al., 2015[15]; Kamio et al., 2014[17]; Martinez et al., 2014[20]; Okuda et al., 2016[22]; Raghu et al., 2012[24]; Sakamoto et al., 2015[31]; Tomioka et al., 2005[36]).

### Risk of bias

**Figure 2:**
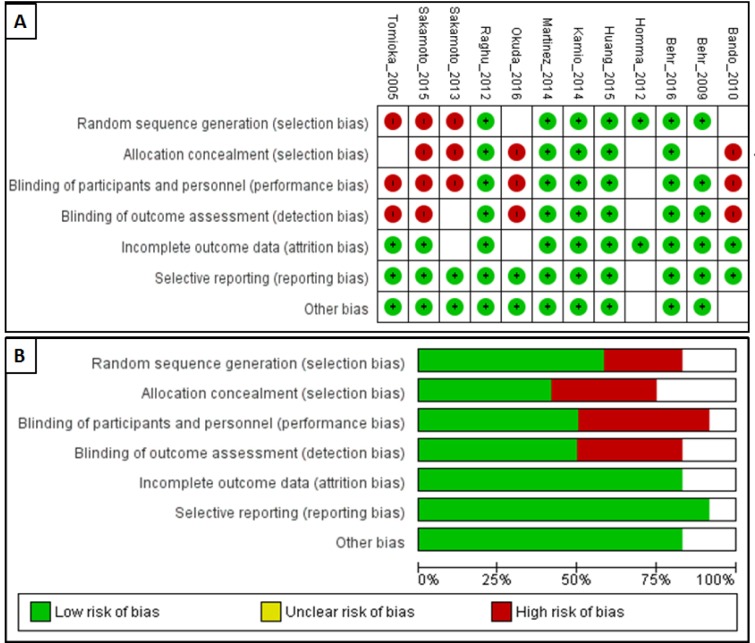
Risk of bias graph of included trials: Review authors' judgments about each risk of bias item for each included study (A) and review authors' judgments about each risk of bias item presented as percentages across all included studies (B).

**Figure 3:**
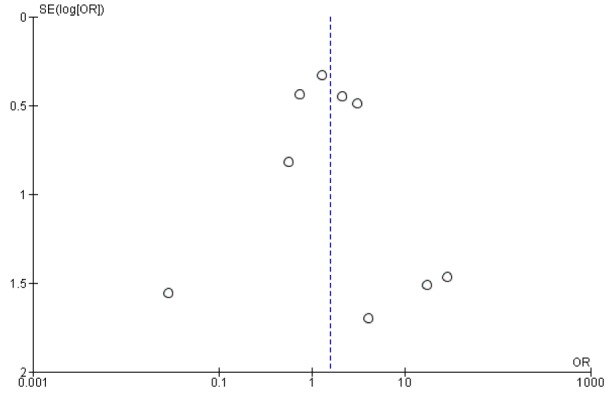
Publication bias analysis by using funnel plot

The ROB assessment for the included studies is presented in Figure 2(Fig. 2). Of the 12 studies identified for analysis in this study, six were classified as high quality (Behr et al., 2009[6], 2016[5]; Huang et al., 2015[15]; Kamio et al., 2014[17]; Martinez et al., 2014[20]; Raghu et al., 2012[24]) and six as low quality (Bando et al., 2010[3]; Homma et al., 2012[14]; Okuda et al., 2016[22]; Sakamoto et al., 2013[30], 2015[31]; Tomioka et al., 2005[36]). However, adverse effects assessing of funnel plots for subgroup analysis revealed an asymmetric distribution, suggesting publication bias (Figure 3(Fig. 3)).

Downs and Black scoring were used to evaluate the quality of the studies. Study quality was variable. The overall quality of study reporting was good, external validity was low, internal validity was better amongst studies (Table 2(Tab. 2)). A heat map of an overview of the quality of the studies determined using Downs and Black scoring system is provided in Supplementary material.

### Conflicting evidence

Many antioxidants (including NAC and PC-SOD) have recently received increased attention as a novel treatment for IPF. A number of studies have evaluated the efficacy and safety of this antioxidant for the treatment of IPF, but with conflicting results (Bando et al., 2010[3]; Behr et al., 2009[6], 2016[5]; Homma et al., 2012[14]; Huang et al., 2015[15]; Kamio et al., 2014[17]; Martinez et al., 2014[20]; Okuda et al., 2016[22]; Raghu et al., 2012[24]; Sakamoto et al., 2013[30], 2015[31]; Tomioka et al., 2005[36]). Several studies found that NAC treatment had a beneficial effect on lung function in IPF patients and significantly delayed disease progression (Behr et al., 2009[6]; Homma et al., 2012[14]; Huang et al., 2015[15]; Okuda et al., 2016[22]; Sakamoto et al., 2013[30], 2015[31]) whereas few studies reported that NAC treatment had no benefit and did not significantly improve the lung function of patients with IPF (Bando et al., 2010[3]; Behr et al., 2016[5]; Martinez et al., 2014[20]; Raghu et al., 2012[24]). A study by Tomioka et al. (2005[36]) showed that N-acetylcysteine aerosol treatment might delay disease progression, but did not improve pulmonary function or QoL. However, PC-SOD treatment had a beneficial effect on serum markers in IPF patients with severe respiratory dysfunction (Kamio et al., 2014[17]). As low statistical power may limit the interpretability of the findings thus results of all of these trials (Bando et al., 2010[3]; Behr et al., 2009[6], 2016[5]; Homma et al., 2012[14]; Huang et al., 2015[15]; Kamio et al., 2014[17]; Martinez et al., 2014[20]; Okuda et al., 2016[22]; Raghu et al., 2012[24]; Sakamoto et al., 2013[30], 2015[31]; Tomioka et al., 2005[36]) should be interpreted with caution.

### Outcomes

Because of heterogeneity in the studies, we used a random-effects model for the analysis of ΔFVC (L), ΔFVC (%), Δ%VC, Δ%DLco, Δ6MWT, adverse events and death (Table 3(Tab. 3)). 

#### Effect of antioxidant on Δ%VC

**Figure 4:**
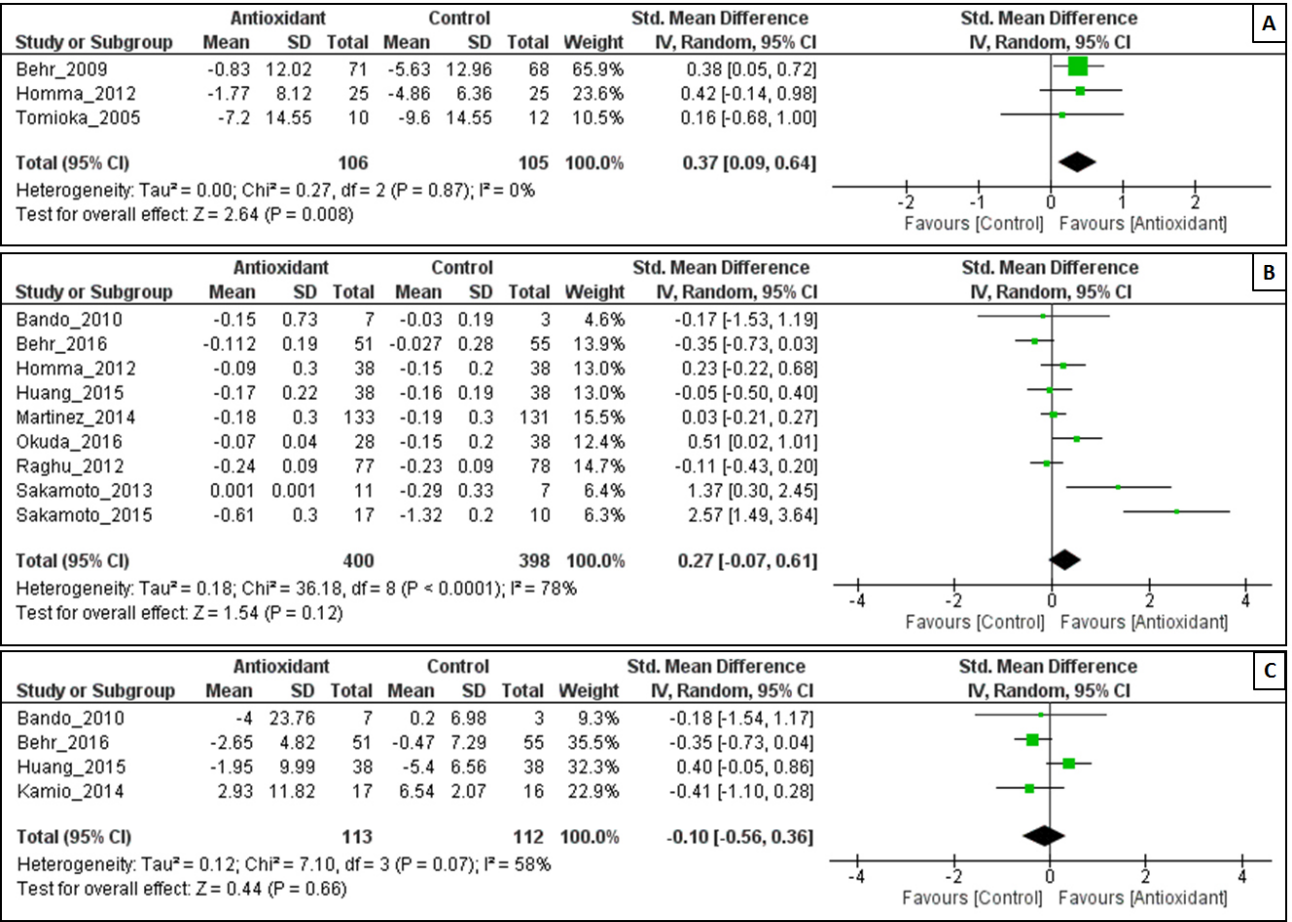
Forest plot evaluating effects of antioxidants on Δ%VC (A), ΔFVC (L) (B) and ΔFVC (%) (C) compared with control group by using random-effects model

As shown in Figure 4A(Fig. 4), antioxidant combination resulted in a significant change in %VC, with an overall effect size (Z) of 2.64 (p = 0.008) and no evidence of heterogeneity was observed (I^2^ = 0 %; P = 0.87). The decrease in %VC was significantly less in the antioxidant group than in the control group (SMD = 0.37, 95% CI: 0.09 to 0.64; P = 0.008).

#### Effect of antioxidant on ΔFVC

The efficacy of antioxidant treatment in reduction of FVC (L) lost its statistical significance (Z = 1.54; P = 0.12). There was heterogeneity between the studies (I^2^ = 78 %; p = 0.0001). There were no statistically significant differences in FVC (L) in the antioxidant group than in the control group (SMD = 0.27, 95% CI:-0.07 to 0.61; P = 0.12) (Figure 4B(Fig. 4)).

The intervention of antioxidant use did not associate with a significant decrease in ΔFVC (%) (Z = 0.44; P = 0.66), however it has acceptable heterogeneity among the studies (I^2^ = 58 %; P = 0.07). There were no statistically significant differences in FVC (%) in the antioxidant group than in the control group (SMD = -0.10, 95% CI:-0.56 to 0.36; P = 0.66) (Figure 4C(Fig. 4)).

#### Effect of antioxidant on 6MWT 

**Figure 5:**
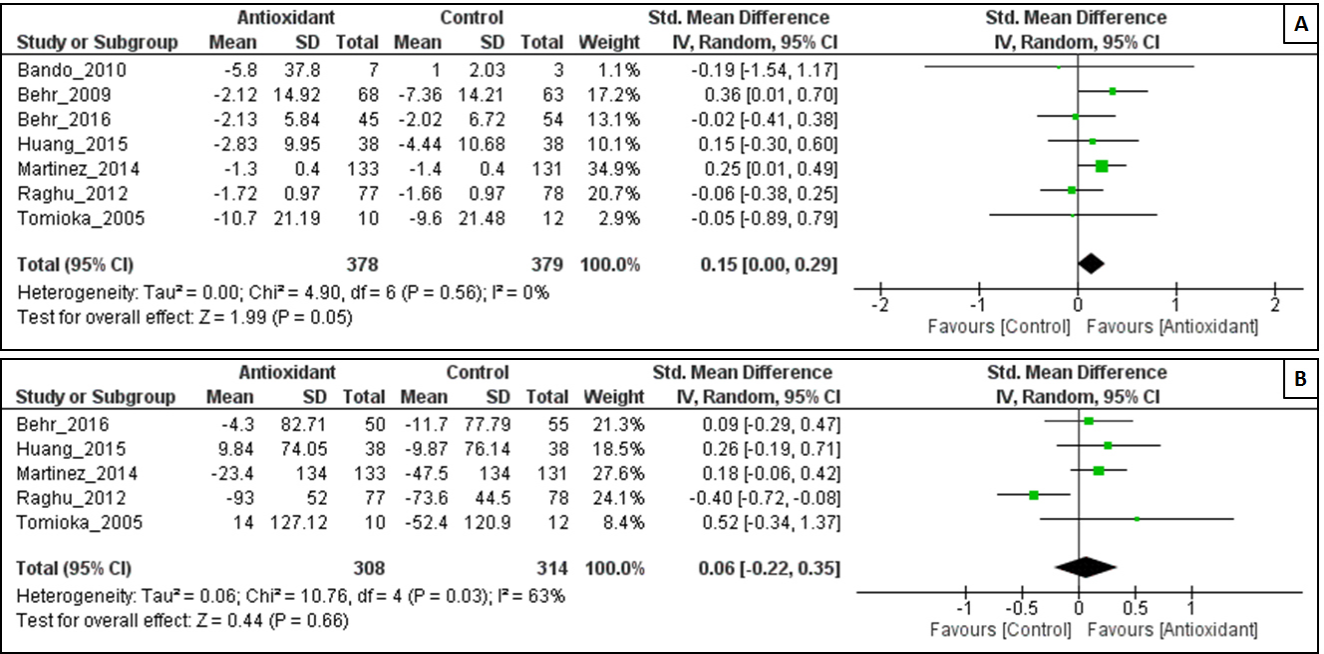
Forest plot evaluating effects of antioxidants on Δ%DLco (A) and Δ6MWT (B) compared with control group by using random-effects model

The intervention of antioxidant used, was associated with a less significant decline in 6MWT (Z = 0.53; P = 0.59), with substantial heterogeneity among the studies (I^2^ = 63 %; P = 0.03). The decline in 6MWT was significantly less in the antioxidant group than in the control group (SMD = -0.04, 95% CI:-0.11 to 0.20; P = 0.59) (Figure 5A(Fig. 5)).

#### Effect of antioxidant on Δ%DLco

As shown in Figure 5B(Fig. 5), antioxidant combination resulted in a significant change in Δ%DLco, with an overall effect size (Z) of 1.99 (p = 0.05) and no evidence of heterogeneity was observed (I^2^ = 0 %; P = 0.56). The change in Δ%DLco was significantly less in the antioxidant group than in the control group (SMD = 0.15, 95% CI: 0.00 to 0.29; P = 0.05).

#### Adverse effects of antioxidants

**Figure 6:**
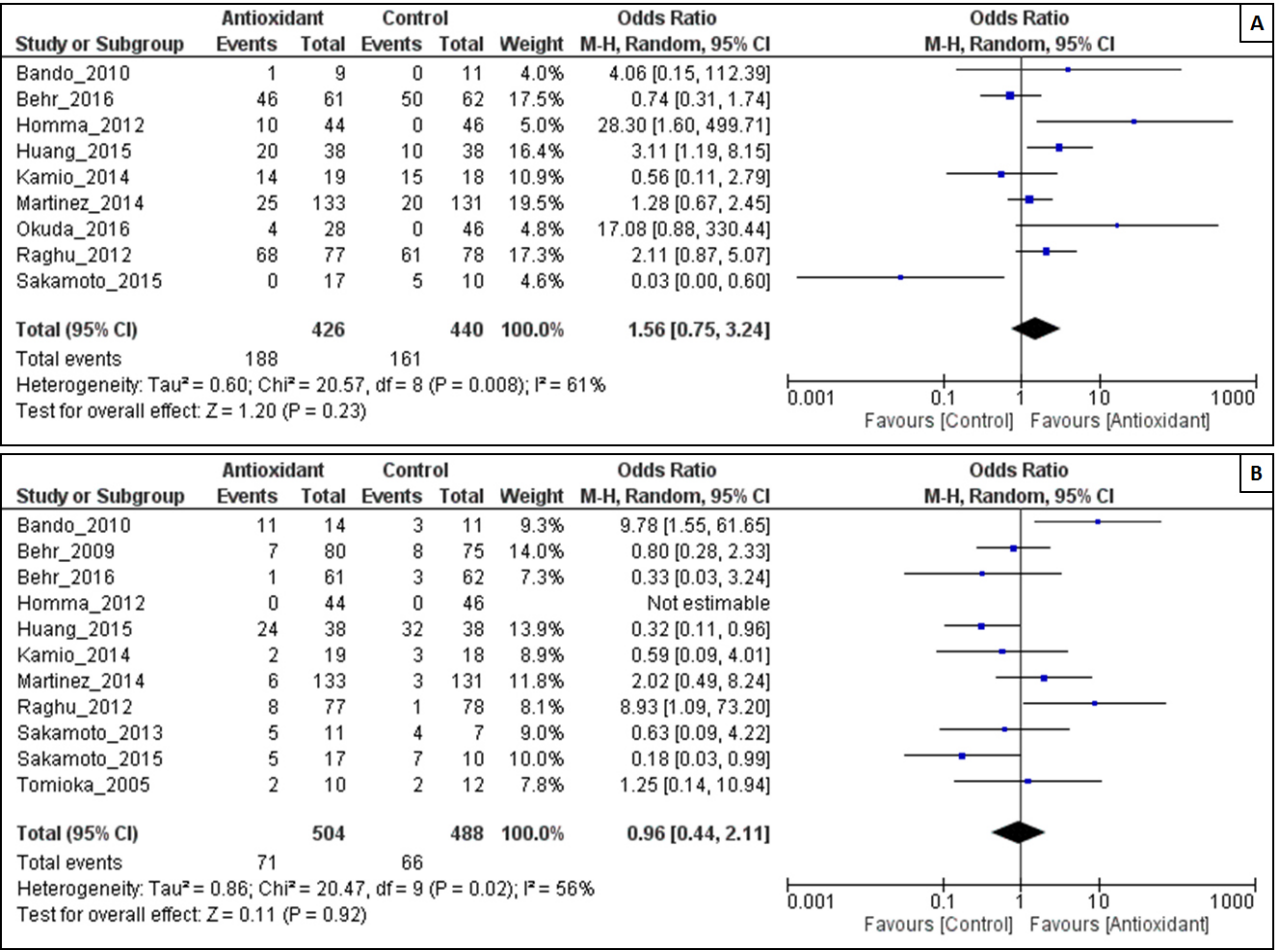
Forest plot evaluating effects of antioxidants on the rate of adverse events (A) and rate of death (B) compared with the control group by using a random-effects model

We also performed meta-analyses regarding any adverse effects of antioxidants mentioned in the included studies. There were also no statistically significant differences in the occurrence of adverse events (OR = 1.56, 95% CI: 0.75 to 3.24; P = 0.23) with substantial heterogeneity among the studies (I^2^ = 61 %; P = 0.0008) (Figure 6A(Fig. 6)).

#### Effect of antioxidant on rate of death

The intervention of antioxidant used, did not associate with a significant death (Z = 0.11; P = 0.92), however it has acceptable heterogeneity among the studies (I^2^ = 56 %; P = 0.02). There were no statistically significant differences in rate of death in the antioxidant group than in the control group (OR = 0.96, 95% CI: 0.44 to 2.11; P = 0.92) (Figure 6B(Fig. 6)).

## Discussion

An array of factors that are associated with IPF such as prognostic factors, stage of IPF, complications, as well as comorbidities as these needs to be addressed before treating IPF patients. Various non-pharmacological treatments such as respiratory rehabilitation, home oxygen therapy, cell and gene therapy, lung transplantation, and palliative care have been reported to produce beneficial effect up to some extent in IPF patients (Chan et al., 2013[8]; Xaubet et al., 2013[38]). However, these treatment strategies failed to address the issues such as risk factors that may aggravate IPF as well as unable to provide symptomatic relief (Richeldi et al., 2003[29]). Hence, pharmacological interventions acquired more attention as treatment strategies for IPF (Bando et al., 2010[3]; Behr et al., 2009[6], 2016[5]; Chan et al., 2013[8]; Demedts et al., 2005[11]; Homma et al., 2012[14]; Jiang et al., 2012[16]; Martinez et al., 2014[20]; Taniguchi et al., 2010[34]; Tomioka et al., 2005[36]).

According to American Thoracic Society-European Respiratory Society, the recommended therapy for IPF consists of prednisone and azathioprine (American Thoracic Society, 2000[1]). However, there is limited evidence supporting prednisone for cough suppression in IPF. Another agent that might be useful as a therapy for treatment of IPF is pirfenidone, but its effect as a single agent is not well established (Azuma et al., 2005[2]).

Moreover, these agents also associated with an array of side effects such as physical deconditioning after administration of prednisone and myelotoxic effects with azathioprine. However, there is increasing evidence suggesting that oxidant-mediated alveolar epithelial cell injury along with abnormal fibroblast response is responsible for generation and maintenance of IPF (Chan et al., 2013[8]). With this view, administration of agents with antioxidant potential might be useful for the treatment of IPF. Hence, in this systematic review and meta-analysis, we evaluated the efficacy of antioxidant in the management of patients with IPF. 

Meta-analysis is an important statistical tool and essential part of a systematic review which aids clinical decision-making. An array of studies revealed that antioxidant therapy showed a potent effect against IPF. However, the clinical implication remains sporadic worldwide. The trial of Kamio et al. (2014[17]) showed that treatment with antioxidants improve the levels of serum markers in patients with severe respiratory dysfunction that improved the QoLin IPF patients (Kamio et al., 2014[17]) whereas Bando et al. (2010[3]) showed that QoL of IPF patients worsened with antioxidant therapy (Bando et al., 2010[3]). Thus, these conflicts led us to investigate the effectiveness of antioxidants in IPF further by meta-analyzing the data collated from these studies. Previous systematic review and meta-analyses of antioxidant therapy in the management of IPF were unable to forge a conclusive body of evidence due to few RCTs which reported the effect of antioxidant treatments on IPF.

The role of antioxidants in the treatment of IPF has been studied by various researchers (Bando et al., 2010[3]; Behr et al., 2009[6], 2016[5]; Homma et al., 2012[14]; Huang et al., 2015[15]; Kamio et al., 2014[17]; Martinez et al., 2014[20]; Okuda et al., 2016[22]; Raghu et al., 2012[24]; Sakamoto et al., 2013[30], 2015[31]; Tomioka et al., 2005[36]) and these studies were included in present systematic reviews and meta-analysis. Effect of antioxidant such as N-acetylcysteine (NAC) has been studied as a single agent or in combination, with variable results (Bando et al., 2010[3]; Behr et al., 2009[6], 2016[5]; Homma et al., 2012[14]; Huang et al., 2015[15]; Martinez et al., 2014[20]; Okuda et al., 2016[22]; Raghu et al., 2012[24]; Sakamoto et al., 2013[30], 2015[31]; Tomioka et al., 2005[36]) where lecithinized superoxide dismutase (PC-SOD) have been studied as a single agent (Kamio et al., 2014[17]). Previous reports showed that there was no significant impact on Δ%DLco by treatment with single antioxidant therapy (SMD=0.12, 95% CI: -0.06-0.30; P=0.18) (Sun et al., 2016[33]), whereas use of a combination of antioxidant with other therapies resulted in a significant improvement in Δ%DLco (SMD = 0.15, 95% CI: 0.00 to 0.29; P = 0.05). Thus, treatment with a combination of antioxidants may be beneficial for amelioration the alteration in DLco rather than treating with single antioxidant. 

On the basis of results of the present meta-analysis, it can be supported that antioxidants play a supportive role in the improving QoL of IPF patients reflected by providing the benefits in terms of VC and DLco. The result of the present meta-analysis is in contrast with the findings of some previous clinical trials which advocate antioxidants unlikely to be beneficial in IPF (Bando et al., 2010[3]; Behr et al., 2016[5]; Martinez et al., 2014[20]). Alteration in VC and DLco are the hallmarks of IPF and their amelioration by antioxidant proves its therapeutic potential for IPF treatment. Findings of the present meta-analysis showed that antioxidant exerts a beneficial effect in terms of VC and DLco thus support its potential of ameliorating IPF.

Antioxidants are widely used for the treatment of an array of diseases with or without the advice of their physician. However, they may associate with serious adverse effects. The results of our meta-analysis showed that antioxidants failed to provide discernible benefits exhibited by reduced adverse events. As antioxidant treatment is associated with an array of adverse events (such as a headache, nausea, constipation, allergies, and diarrhea), thus number of patients were withdrawn from studies, and we generated the results by extrapolating these dropouts. Thus, it was not surprising that adverse events were more in the antioxidant than the placebo group. Furthermore, these adverse events were minimal and manageable with additional supportive treatments. Although antioxidants were associated with mild adverse events but a subsequent meta-analysis of eleven studies showed that it did not associate with a significant mortality (OR = 0.96, 95% CI: 0.44 to 2.11; P = 0.92).

Using statistical methods, we analyzed the results of various studies to overcome the limits of earlier studies where single antioxidant was used for the treatment of IPF. Results of present study evidence that use of antioxidants monotherapy showed promising efficacy in IPF patients. However, results did not show any beneficial effect on changes in predicted carbon monoxide diffusing capacity. Interestingly, when we focused on the combined antioxidant trials, a strong association of antioxidants with predicted carbon monoxide diffusing capacity in patients with IPF was observed. 

This meta-analysis has potential limitations which include the use of significant clinical heterogeneity and low statistical power. We applied random-effects model to address residual heterogeneity in subsequent subgroup analysis. Furthermore, the studies were grossly disparate in their use of antioxidant agents, high dropout rates, duration of follow-ups, and dosing regimens. Also, some studies included in this meta-analysis lacked necessary data, thus hindering the assessment of some meaningful end points. In the light of these considerations, additional high-quality randomized controlled trials (RCTs) are needed to add weight to the analysis and formulate robust evidence-based clinical recommendations.

In conclusion, results of the present meta-analysis indicate that oxidative stress plays a vital role in the pathophysiology of IPF, and combined antioxidant therapy may provide comparatively higher clinical improvements (e.g. VC, and DLco improvements) compared to monotherapy. However, further research in subgroups of patients with IPF who obtain optimal benefit from combined antioxidant therapy needs investigational credence. 

## Implications for Research

The nature of outline and presentation of the studies was mostly poor. Methodology as well as the approach was frequently and not entirely depicted. The studies were, for the most part, underpowered to demonstrate any advantage that might be available over other treatments. In future, it will be imperative that the entry characteristics of the patient are well characterized, and if possible comparable between trials. There should be well-defined inclusion, exclusion criteria and primary outcome measures used. As well as, a more detailed description of adverse events is needed.

## Supplementary Material

Supplementary material

## Figures and Tables

**Table 1:**
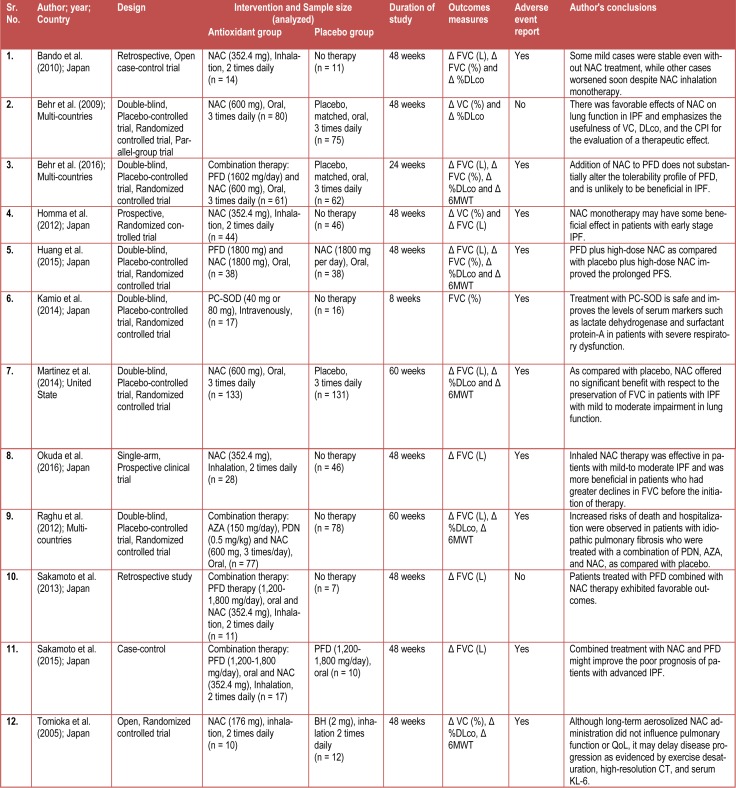
Characteristics of included studies

**Table 2:**
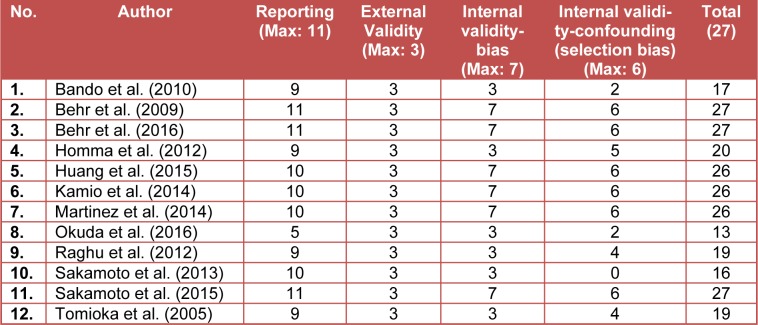
Review of the quality of studies determined using Downs and Black scoring system

**Table 3:**
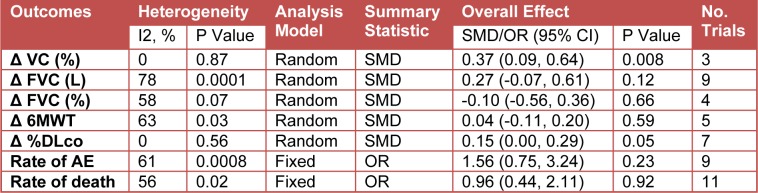
Clinical outcomes of antioxidant group compared with control group

## References

[1] American Thoracic Society (2000). Idiopathic pulmonary fibrosis: diagnosis and treatment. International consensus statement. American Thoracic Society (ATS), and the European Respiratory Society (ERS). Am J Respir Crit Care Med.

[2] Azuma A, Nukiwa T, Tsuboi E, Suga M, Abe S, Nakata K (2005). Double-blind, placebo-controlled trial of pirfenidone in patients with idiopathic pulmonary fibrosis. Am J Respir Crit Care Med.

[3] Bando M, Hosono T, Mato N, Nakaya T, Yamasawa H, Ohno S (2010). Long-term efficacy of inhaled N-acetylcysteine in patients with idiopathic pulmonary fibrosis. Intern Med.

[4] Beeh KM, Beier J, Haas IC, Kornmann O, Micke P, Buhl R (2002). Glutathione deficiency of the lower respiratory tract in patients with idiopathic pulmonary fibrosis. Eur Respir J.

[5] Behr J, Bendstrup E, Crestani B, Gunther A, Olschewski H, Skold CM (2016). Safety and tolerability of acetylcysteine and pirfenidone combination therapy in idiopathic pulmonary fibrosis: a randomised, double-blind, placebo-controlled, phase 2 trial. Lancet Respir Med.

[6] Behr J, Demedts M, Buhl R, Costabel U, Dekhuijzen RP, Jansen HM (2009). Lung function in idiopathic pulmonary fibrosis - extended analyses of the IFIGENIA trial. Respir Res.

[7] Cantin AM, Hubbard RC, Crystal RG (1989). Glutathione deficiency in the epithelial lining fluid of the lower respiratory tract in idiopathic pulmonary fibrosis. Am Rev Respir Dis.

[8] Chan AL, Rafii R, Louie S, Albertson TE (2013). Therapeutic update in idiopathic pulmonary fibrosis. Clin Rev Allergy Immunol.

[9] Cochrane (2008). Cochrane handbook for systematic reviews of interventions 5.0.1.

[10] Cottin V, Maher T (2015). Long-term clinical and real-world experience with pirfenidone in the treatment of idiopathic pulmonary fibrosis. Eur Respir Rev.

[11] Demedts M, Behr J, Buhl R, Costabel U, Dekhuijzen R, Jansen HM (2005). High-dose acetylcysteine in idiopathic pulmonary fibrosis. N Engl J Med.

[12] du Bois RM (2012). An earlier and more confident diagnosis of idiopathic pulmonary fibrosis. Eur Respir Rev.

[13] Gribbin J, Hubbard RB, Le Jeune I, Smith CJ, West J, Tata LJ (2006). Incidence and mortality of idiopathic pulmonary fibrosis and sarcoidosis in the UK. Thorax.

[14] Homma S, Azuma A, Taniguchi H, Ogura T, Mochiduki Y, Sugiyama Y (2012). Efficacy of inhaled N-acetylcysteine monotherapy in patients with early stage idiopathic pulmonary fibrosis. Respirology.

[15] Huang H, Dai HP, Kang J, Chen BY, Sun TY, Xu ZJ (2015). Double-blind randomized trial of pirfenidone in chinese idiopathic pulmonary fibrosis patients. Medicine (Baltimore).

[16] Jiang C, Huang H, Liu J, Wang Y, Lu Z, Xu Z (2012). Adverse events of pirfenidone for the treatment of pulmonary fibrosis: a meta-analysis of randomized controlled trials. PLoS ONE.

[17] Kamio K, Azuma A, Ohta K, Sugiyama Y, Nukiwa T, Kudoh S (2014). Double-blind controlled trial of lecithinized superoxide dismutase in patients with idiopathic interstitial pneumonia - short term evaluation of safety and tolerability. BMC Pulm Med.

[18] Lee JS, Ryu JH, Elicker BM, Lydell CP, Jones KD, Wolters PJ (2011). Gastroesophageal reflux therapy is associated with longer survival in patients with idiopathic pulmonary fibrosis. Am J Respir Crit Care Med.

[19] Luppi F, Spagnolo P, Cerri S, Richeldi L (2012). The big clinical trials in idiopathic pulmonary fibrosis. Curr Opin Pulm Med.

[20] Martinez FJ, de Andrade JA, Anstrom KJ, King TE, Jr, Raghu G, Idiopathic Pulmonary Fibrosis Clinical Research Network (2014). Randomized trial of acetylcysteine in idiopathic pulmonary fibrosis. N Engl J Med.

[21] Nalysnyk L, Cid-Ruzafa J, Rotella P, Esser D (2012). Incidence and prevalence of idiopathic pulmonary fibrosis: review of the literature. Eur Respir Rev.

[22] Okuda R, Matsushima H, Oba T, Kawabe R, Matsubayashi M, Amano M (2016). Efficacy and safety of inhaled N-acetylcysteine in idiopathic pulmonary fibrosis: A prospective, single-arm study. Respir Investig.

[23] Pinheiro GA, Antao VC, Wood JM, Wassell JT (2008). Occupational risks for idiopathic pulmonary fibrosis mortality in the United States. Int J Occup Environ Health.

[24] Raghu G, Anstrom KJ, King TE Jr, Lasky JA, Martinez FJ, Idiopathic Pulmonary Fibrosis Clinical Research Network (2012). Prednisone, azathioprine, and N-acetylcysteine for pulmonary fibrosis. N Engl J Med.

[25] Raghu G, Chen SY, Hou Q, Yeh WS, Collard HR (2016). Incidence and prevalence of idiopathic pulmonary fibrosis in US adults 18-64 years old. Eur Respir J.

[26] Raghu G, Chen SY, Yeh WS, Maroni B, Li Q, Lee YC (2014). Idiopathic pulmonary fibrosis in US Medicare beneficiaries aged 65 years and older: incidence, prevalence, and survival, 2001-11. Lancet Respir Med.

[27] Raghu G, Collard HR, Egan JJ, Martinez FJ, Behr J, Brown KK (2011). An official ATS/ERS/JRS/ALAT statement: idiopathic pulmonary fibrosis: evidence-based guidelines for diagnosis and management. Am J Respir Crit Care Med.

[28] Raghu G, Rochwerg B, Zhang Y, Garcia CA, Azuma A, Behr J (2015). An Official ATS/ERS/JRS/ALAT Clinical Practice Guideline: Treatment of Idiopathic Pulmonary Fibrosis. An Update of the 2011 Clinical Practice Guideline. Am J Respir Crit Care Med.

[29] Richeldi L, Davies HR, Ferrara G, Franco F Corticosteroids for idiopathic pulmonary fibrosis. Cochrane Database Syst Rev. 2003:CD002880.

[30] Sakamoto S, Itoh T, Muramatsu Y, Satoh K, Ishida F, Sugino K (2013). Efficacy of pirfenidone in patients with advanced-stage idiopathic pulmonary fibrosis. Intern Med.

[31] Sakamoto S, Muramatsu Y, Satoh K, Ishida F, Kikuchi N, Sano G (2015). Effectiveness of combined therapy with pirfenidone and inhaled N-acetylcysteine for advanced idiopathic pulmonary fibrosis: a case-control study. Respirology.

[32] Stewart LA, Clarke M, Rovers M, Riley RD, Simmonds M, Stewart G (2015). Preferred reporting items for a systematic review and meta-analysis of individual participant data: The PRISMA-IPD Statement. JAMA.

[33] Sun T, Liu J, Zhao de W (2016). Efficacy of N-acetylcysteine in idiopathic pulmonary fibrosis: a systematic review and meta-analysis. Medicine (Baltimore).

[34] Taniguchi H, Ebina M, Kondoh Y, Ogura T, Azuma A, Suga M (2010). Pirfenidone in idiopathic pulmonary fibrosis. Eur Respir J.

[35] Taskar VS, Coultas DB (2006). Is idiopathic pulmonary fibrosis an environmental disease?. Proc Am Thorac Soc.

[36] Tomioka H, Kuwata Y, Imanaka K, Hashimoto K, Ohnishi H, Tada K (2005). A pilot study of aerosolized N-acetylcysteine for idiopathic pulmonary fibrosis. Respirology.

[37] Xaubet A, Ancochea J, Blanquer R, Montero C, Morell F, Rodriguez Becerra E (2003). Arch Bronconeumol.

[38] Xaubet A, Ancochea J, Bollo E, Fernandez-Fabrellas E, Franquet T, Molina-Molina M (2013). Guidelines for the diagnosis and treatment of idiopathic pulmonary fibrosis. Sociedad Espanola de Neumologia y Cirugia Toracica (SEPAR), Research Group on Diffuse Pulmonary Diseases. Arch Bronconeumol.

