# Supplementary material for: Efficacy of antioxidant in idiopathic pulmonary fibrosis: A systematic review and meta-analysis
Source: EXCLI J. 2016 Nov 7;15:636–51. doi: 10.17179/excli2016-619 (PMC5225735; doi:10.17179/excli2016-619)
Supplement: Supplementary material [file EXCLI-15-636-s-001.pdf]

EXCLI Journal 2016;15:636-651 – ISSN 1611-2156
Received: September 01, 2016, accepted: October 15, 2016, published: November 07, 2016

Supplementary material to:

EFFICACY OF ANTIOXIDANT IN IDIOPATHIC PULMONARY FIBROSIS: A SYSTEMATIC REVIEW AND META-ANALYSIS

Amit D. Kandhare, Anwesha Mukherjee, Pinaki Ghosh, Subhash L. Bodhankar*

Department of Pharmacology, Poona College of Pharmacy, Bharati Vidyapeeth Deemed University, Erandwane, Paud Road, Pune-411 038, India

* Corresponding author: Dr. Subhash L. Bodhankar, Professor, and Head, Department of Pharmacology, Poona College of Pharmacy, Bharati Vidyapeeth Deemed University, Erandwane, Paud Road, Pune-411038, Maharashtra, India. Phone: +91-20-25437237, Fax: +91-20-25439383; E-mail: drslbodh@gmail.com

http://dx.doi.org/10.17179/excli2016-619

This is an Open Access article distributed under the terms of the Creative Commons Attribution License (http://creativecommons.org/licenses/by/4.0/).

S. No.	Study ID	Country	Downs and Black scoring system																														
			Reporting										Mean	External validity			Mean	Internal validity - bias							Mean	Internal validity -confounding (selection bias)						Mean	Total
			1 Is the hypothesis/ objective of the study clearly described?	2 Are the main outcomes to be measured clearly described in the introduction or methods section?	3 Are the characteristics of the patients included in the study clearly described?	4 Are the interventions of interest clearly described?	5* Are the distributions of principal confounders in each group of subjects to be compared clearly described?	6 Are the main findings of the study clearly described?	7 Does the study provide estimates of the random variability in the data for the main outcomes?	8 Have all important adverse events that may be a consequence of the intervention been reported?	9 Have the characteristics of patients lost to follow-up been described?	10 Have actual probability values been reported (eg. 0.035 rather than <0.05) for the main outcomes except where the probability value is less than 0.001?		11 Were the subjects asked to participate in the study representative of the entire population from which they were recruited?	12 Were those subjects who were prepared to participate representative of the entire population from which they were recruited?	13 Were the staff, places and facilities where the patients were treated representative of the treatment the majority of the patients receive?		14 Was an attempt made to blind study subjects to the intervention they received?	15 Was an attempt made to blind those measuring the main outcomes of the intervention?	16 If any results of the study were based on "data dredging" was this made clear?	17 In trials and cohort studies, do the analyses adjust for different lengths of follow up of patients, or in case control studies, is the time period between the intervention and outcome the same for cases and controls?	18 Were the statistical tests used to assess the main outcomes appropriate?	19 Was compliance with the interventions reliable?	20 Were the main outcomes measures used accurate (valid and reliable)?		21 Were the patients in different intervention groups (trials and cohort studies) or were the cases and controls (case-control studies) recruited from the same population?	22 Were study subjects in different intervention groups (trials and cohort studies) or were the cases and controls (case-control studies) recruited over the same period of time?	23 Were study subjects randomised to intervention groups?	24 Was the randomised intervention assignment concealed from both patients and healthcare staff until recruitment was complete and irrevocable?	25 Was there adequate adjustment for confounding in the analyses from which the main findings were drawn?	26 Were losses of patients to follow-up taken into account?		
1	Bando_2010	Japan	1	1	1	1	1	1	1	0	1	1	**9**	1	1	1	**3**	0	0	0	0	1	1	1	**3**	1	1	0	0	0	0	**2**	**17**
2	Behr_2009	Multi-countries	1	1	1	1	2	1	1	1	1	1	**11**	1	1	1	**3**	1	1	1	1	1	1	1	**7**	1	1	1	1	1	1	**6**	**27**
3	Behr_2016	Multi-countries	1	1	1	1	2	1	1	1	1	1	**11**	1	1	1	**3**	1	1	1	1	1	1	1	**7**	1	1	1	1	1	1	**6**	**27**
4	Homma_2012	Japan	1	1	1	1	1	1	1	1	0	1	**9**	1	1	1	**3**	0	0	0	0	1	1	1	**3**	1	1	1	1	1	0	**5**	**20**
5	Huang_2015	China	1	1	1	1	1	1	1	1	1	1	**10**	1	1	1	**3**	1	1	1	1	1	1	1	**7**	1	1	1	1	1	1	**6**	**26**
6	Kamio_2014	Japan	1	1	1	1	1	1	1	1	1	1	**10**	1	1	1	**3**	1	1	1	1	1	1	1	**7**	1	1	1	1	1	1	**6**	**26**
7	Martinez_2014	United State	1	1	1	1	1	1	1	1	1	1	**10**	1	1	1	**3**	1	1	1	1	1	1	1	**7**	1	1	1	1	1	1	**6**	**26**
8	Okuda_2016	Japan	1	1	1	1	1	1	1	1	1	1	**10**	1	1	1	**3**	0	0	0	0	1	1	1	**3**	0	0	0	0	0	0	**0**	**16**
9	Raghu_2012	Japan	1	1	1	1	2	1	1	1	1	1	**11**	1	1	1	**3**	1	1	1	1	1	1	1	**7**	1	1	1	1	1	1	**6**	**27**
10	Sakamoto_2013	Japan	1	1	1	1	1	1	1	0	1	1	**9**	1	1	1	**3**	0	0	0	0	1	1	1	**3**	1	1	0	0	1	1	**4**	**19**
11	Sakamoto_2015	Japan	1	1	0	0	0	1	1	0	0	1	**5**	1	1	1	**3**	0	0	0	0	1	1	1	**3**	1	1	0	0	0	0	**2**	**13**
12	Tomioka_2005	Japan	1	1	1	1	1	1	1	0	1	1	**9**	1	1	1	**3**	0	0	0	0	1	1	1	**3**	1	1	1	1	0	0	**4**	**19**
